# Abnormal Pre-Participation Cardiac Screening in Athletes: Can an Athlete Train While Waiting for a “Diagnosis”?

**DOI:** 10.1016/j.jacadv.2025.101943

**Published:** 2025-07-07

**Authors:** Raghav T. Bhatia, Sanjay Sharma, Michael Papadakis, Peter N. Dean, Hamish MacLachlan, Aneil Malhotra, Katie Stewart, Eugene H. Chung, Jonathan H. Kim, Matthew W. Martinez, ACC Sports and Exercise Council

**Affiliations:** a Department of Cardiology, Hull University Teaching Hospitals NHS Trust, Kingston-upon-Hull, United Kingdom; b Cardiovascular Clinical Academic Group and Cardiology Research Centre, St. George's University of London, St. George's University Hospitals NHS Foundation Trust, London, United Kingdom; c Department of Pediatrics, Division of Pediatric Cardiology, University of Virginia, Charlottesville, Virginia, USA; d Institute of Sport, Manchester Metropolitan University and University of Manchester, Manchester, United Kingdom; e Cardiovascular Performance Program, Massachusetts General Hospital, Boston, Massachusetts, USA; f Emory Clinical Cardiovascular Research Institute, Emory School of Medicine, Atlanta, Georgia, USA; g Morristown Medical Center, Atlantic Health System, Morristown, New Jersey, USA

**Keywords:** electrocardiogram, prevention, screening, sudden cardiac death

**Figure:**
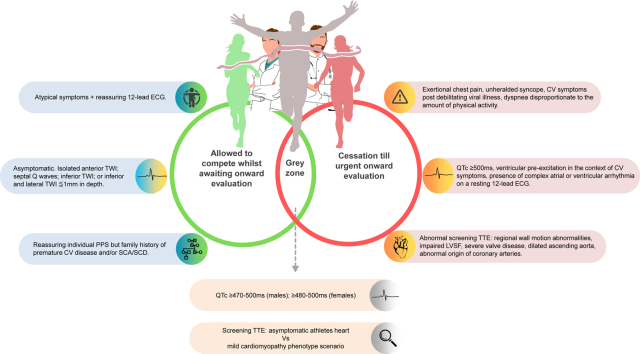

The quest for effective pre-participation screening in young athletes is centered around identifying largely quiescent conditions, tailoring risk stratification, and preventing exercise-related sudden cardiac arrest (SCA) or sudden cardiac death (SCD). Contemporary practice simultaneously emphasizes the role of shared decision-making (SDM), particularly where abnormalities are suspected or confirmed following comprehensive evaluations.

Despite debates regarding the evidence and optimal strategies for screening, most elite sporting organizations recommend regular cardiac screening across various age groups and sporting disciplines. Furthermore, extensive research has reduced false positive findings over the past 2 decades. This progress and reassurance at the time of screening has primarily been achieved through the refinement of electrocardiographic (ECG) criteria, and improvement in our ability to better differentiate between exercise-induced physiological adaptations from pathological conditions via ECG and cardiac imaging.^1^

Variations in professional and elite athlete screening practices exist as a function of the sporting governing body, regional variations in practice, and the availability of resources.^2^, ^3^, ^4^ For example, across North America, the prevailing standard of care continues to be the American Heart Association 14-point screening evaluation tool, with a noticeable shift toward the incorporation of the 12-lead ECG at all levels of sport.^3^^,^^4^ Determining the frequency of screening based on an athlete’s age is also an evolving and topical area as inherited cardiac conditions associated with SCA/SCD in young athletes frequently present in adolescence.^5^^,^^6^

If an abnormality is discovered during pre-participation screening, can the athlete continue to partake in high-intensity training and competitive sport, while awaiting confirmatory investigations and risk stratification? This is an integral question that arises at a critical juncture for many athletes. Coupled with this uncertainty is how providers effectively communicate the situation to the athlete, parents or guardians, and other team doctors while arranging further assessments. We acknowledge that this area remains largely devoid of definitive evidence and that clinicians' views and practices may vary based on their experience, level of comfort, the specific circumstances of the young individual, overall risk profile, and medicolegal considerations.

Given these concerns and the inevitability of identifying abnormalities, screening providers must establish a clear plan for managing these young athletes. This includes determining: 1) who will communicate the findings to the athlete; 2) what further testing or consultations are required; and 3) who will conduct them. The goal is to minimize delays between initial screening and definitive diagnosis. The goal is not only to identify abnormalities but also ensure that athletes are not left in diagnostic uncertainty. There must be a clear and timely plan for further evaluation to definitively diagnose or rule out cardiac pathology.

Based on practical experience and consensus recommendations,^2^, ^3^, ^4^ individuals reporting worrisome and “red flag” cardiovascular symptoms, such as exertional chest pain, inappropriate shortness of breath, palpitations associated with presyncope or syncope, or unheralded syncope should be advised to abstain from high-intensity training and competitive sport until further evaluation is completed (Figure 1).

**Figure 1:**
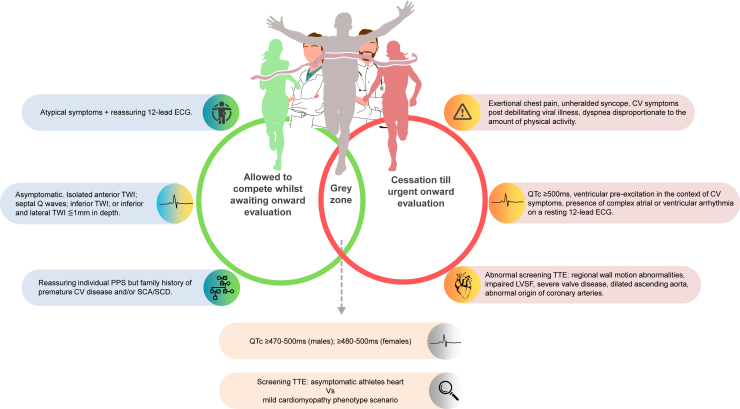
Stratified Approach to Abnormal Pre-Participation Cardiac Screening in Athletes CV = cardiovascular; ECG = electrocardiogram; LVSF = left ventricular systolic function; PPS = pre-participation screening; SCA = sudden cardiac arrest; SCD = sudden cardiac death; TTE = transthoracic echocardiogram; TWI = T-wave inversion.

If screening practices include a resting 12-lead ECG, the findings of a QTc interval ≥500 ms; high burden of ventricular ectopy ≥2 premature ventricular contractions; or marked ST-T repolarization changes suspicious of cardiomyopathy likely warrant a temporary pause in training until the arrhythmogenicity of the underlying substrate is appreciated following a timely comprehensive onward evaluation is completed (Figure 1). If an ECG is performed, a plan for timely downstream evaluation including a transthoracic echocardiogram is needed to identify or exclude pathological substrates. Echocardiograms can identify additional findings the ECG cannot identify such as dilatation of the aortic root or ascending aorta (≥45 mm max dimension) or left ventricular systolic dysfunction (ejection fraction <45%); and anomalous origins of coronary arteries,^7^ as examples. Athletes may receive recommendations to temporarily refrain from engaging in high-intensity training and competitive sports until a comprehensive risk assessment is conducted, particularly in the context of cardiovascular symptoms. It is worth acknowledging the intrinsic link between the quality of echocardiography images on the day and the proficiency of the operator.

To contextualize many of the aforementioned concepts, voluntary cardiac screening initiatives in the United Kingdom, such as those provided by the charity Cardiac Risk in the Young, are open to all individuals aged 14 to 35 years, extending beyond elite athletes. Those deemed high risk after initial screening are advised to avoid competitive or high-intensity exercise until further investigations are completed. Given that some follow-up assessments can take months and vary depending on available expertise, it is crucial that risk assessment and medical guidance are applied equitably across all individuals, from recreational participants to professional athletes. Considering logistical factors and the welfare of young individuals, on-site echocardiography available at such screenings in the community serves as a readily available tool to promptly exclude severe morphological cardiomyopathies, such as those associated with T-wave inversions. It also serves as a pragmatic and integral tool for exercise guidance while awaiting secondary care evaluations and dedicated surveillance strategies.

While clinical circumstances may warrant conservative exercise restrictions after initial abnormal screening tests, with other abnormal or borderline findings, it may be reasonable to allow continued training for the athlete (Figure 1). Some ECG findings deemed abnormal by the contemporary International ECG Recommendations for athletes^1^ may still not be as suspicious for underlying cardiomyopathy. Examples include isolated inferior T-wave inversions, isolated septal (V_1_ and V_2_) q-waves, anterior T-wave inversions (particularly in female athletes), or lateral T-wave inversions no >1 mm in depth. In asymptomatic athletes, while imaging with echocardiography is recommended to fully exclude an underlying cardiomyopathic process, it may be reasonable to allow ongoing training for the athlete as long as the evaluation is expedited. Similarly, athletes who report atypical symptoms may be supported in continuing high-intensity training and competition, with an understanding that they must undergo timely evaluation and further risk stratification. Examples include palpitations not exclusively during high-intensity exercise, atypical chest discomfort also not exclusively during high-intensity exercise, or syncope immediately after completion of high-intensity exercise consistent with exercise-associated collapse due to suboptimal fluid status.

Contemporary research, particularly data from postmortem studies in victims of SCD, often report upon the circumstances of death. Based on age and underlying phenotype, there appears to be a potential link between younger individuals, such as adolescents, with exercise-related sudden death, in contrast to older individuals, who tend to die at rest.^6^^,^^8^ Such observations indicate that the risk of exercise-related sudden death, while awaiting further investigations, is heterogeneous with variations between age groups and potentially underlying conditions. Therefore, physicians may also decide to allow some athletes to continue partaking in high-intensity training and competitive sport throughout the clinical evaluation based on these demographics.

The timing of subsequent evaluations is crucial for athletes advised to abstain from competition until further notice. This situation creates anxiety for the athlete and affects the club and team. Therefore, conducting the necessary investigations promptly by individuals and centers with expertise becomes of utmost importance. During periods of restriction, on a case-by-case basis and following consultation with a sports cardiologist, athletes may be permitted to engage in low to moderate intensity exercise, but only under the supervision of trained medical personnel in the presence of automated external defibrillators and under the guise of a written and rehearsed Emergency Action Plan. This approach provides an option for the athlete to stay active if they wish to do so and prevent significant deconditioning. It is vital to safeguard their physical conditioning as much as possible once they can resume regular training and competition.

Beyond physical health, an athlete’s mental well-being also requires support during this period of medical uncertainty, ensuring overall well-being amid the challenges of an abnormal cardiac assessment.

Cautiously, important parallels may be drawn from emerging literature that suggest exercise may not be as high a risk in individuals with underlying cardiomyopathies, namely hypertrophic cardiomyopathy (HCM). Athletes with HCM who continued to exercise vigorously and did not experience a high rate of death or life-threatening arrhythmias compared to those patients with HCM who continued with moderate exercise or those who were sedentary.^9^ More recent data found a similar result in elite and professional athletes.^10^ However, it is vital to appreciate that the participants were self-selected with a higher-than-average percentage of vigorous exercise and care was delivered by high-volume expert HCM centers. In the context of the initial abnormal pre-participation screening, providing such holistic insights from emerging literature to athletes and caregivers ensures a SDM approach with adequate onward risk stratification and surveillance at its core to safely facilitate return-to-play decision-making.

Clinical decisions may vary across different medicolegal environments, and the responsibility placed on clinicians to balance athlete safety with the potential risks of stopping or allowing continued participation must be carefully navigated. In the modern era of SDM with athletes, this responsibility becomes complex when a definitive diagnosis has yet to be established, and full risk stratification is incomplete. When abnormalities are detected but not yet fully understood, we would advocate clinicians to engage in open, transparent conversations with athletes and their guardians. This includes discussing the limitations of current findings and the uncertainties surrounding their condition. This should include clinicians clearly communicating the potential risks of continued training without a full diagnosis, while also considering the athlete’s desire to remain active. The lack of a definitive diagnosis does not exempt the physician from legal responsibilities, and failure to properly advise could lead to medicolegal repercussions, especially if an adverse event occurs. Importantly, the physician must clearly and comprehensively document these discussions in detail, ensuring that the athlete (and their guardians, if applicable) fully understand the potential risks and the rationale behind any decision. Furthermore, developing written action plans, especially if training is allowed to continue under medical supervision, may help mitigate legal risk. Ultimately, the goal is to balance the best available medical evidence with the athlete’s values and preferences, while minimizing both medical and legal risks. Involving multidisciplinary teams and ensuring timely evaluations is essential to providing the safest path forward for athletes, particularly in the face of uncertainty.

The question will continue to persist—can your athlete exercise, while undergoing further evaluation following an abnormal initial pre-participation screening? In brief, assess the risk at the time of initial review, collaborate with experts, perform timely onward evaluations, and empower athletes in their journey to success. Furthermore, the process of SDM must be viewed as a continuum and incorporated at each step, from the initial encounter in the screening arena, where naturally a degree of uncertainty exists, through to the onward comprehensive evaluation and surveillance.

## Funding support and author disclosures

The authors have reported that they have no relationships relevant to the contents of this paper to disclose.
